# Genetic variants of VDR and CYP2R1 affect BMI independently of serum vitamin D concentrations

**DOI:** 10.1186/s12881-020-01065-3

**Published:** 2020-06-13

**Authors:** Bence Bakos, Balázs Szili, Boglárka Szabó, Péter Horváth, Gyöngyi Kirschner, János P. Kósa, Erzsébet Toldy, Péter Lakatos, Ádám G. Tabák, István Takács

**Affiliations:** 1 Department of Internal Medicine and Oncology, Semmelweis University Faculty of Medicine, 1098 Korányi S. u. 2/a, Budapest, Hungary; 2 Department of Pulmonology, Semmelweis University Faculty of Medicine, Budapest, Hungary; 3 Clinical Chemistry and Immunology Laboratories, SYNLAB Diagnostic Centre, Budapest, Hungary; 4 Department of Epidemiology and Public Health, University College London, London, UK; 5 Department of Public Health, Semmelweis University Faculty of Medicine, Budapest, Hungary

**Keywords:** Vitamin D, BMI, Genetics, VDR, CYP2R1

## Abstract

**Background:**

Vitamin D metabolism and obesity have been linked by several studies, however the reason for this association is unclear. Our objective was to investigate potential correlations between genetic variants in key enzymes of vitamin D metabolism and the body mass index on a representative and random sample of Hungarian adults.

**Methods:**

Altogether 462 severely vitamin D deficient individuals were studied at the end of winter in order to decrease environmental and maximize any relevant genetic effect. Furthermore, participants with lifestyle factors known to affect vitamin D homeostasis were also excluded. We selected 23 target SNPs in five genes that encode key proteins of vitamin D metabolism (NADSYN1, GC, CYP24A1, CYP2R1, VDR).

**Results:**

Variants in 2 genetic polymorphisms; rs2853564 (VDR) and rs11023374 (CYP2R1) showed a significant association with participants‘ BMI. These associations survived further adjustment for total-, free-, or bioactive-25(OH) vitamin D levels, although the variance explained by these 2 SNPS in BMI heterogeneity was only 3.2%.

**Conclusion:**

Our results show two novel examples of the relationship between genetics of vitamin D and BMI, highlighting the potential role of vitamin D hormone in the physiology of obesity.

## Background

The heritability of obesity is theorized to be between 20 and 90% [1–3]. As a potential source of physiologic understanding and therapeutic targets, the genetic background of obesity is one of the main focuses of today’s obesity research [1, 4].

Vitamin D (VitD) homeostasis and vitamin D deficiency have been linked to obesity [5] both in vitro [6–8] also in observational [9–13] and interventional [14–17] studies. However, results so far have been contradictory regarding the genetic determinants of this relationship [18, 19]. A likely connection between vitamin D receptor (VDR) polymorphisms and the risk of obesity has been suggested [20–23], though contradictions still exist even in this regard [24].

A potential cause of these inconsistencies in previous studies might be the result of unmeasured confounding related to heterogeneous environmental factors and varying vitamin D levels within and between study populations. Indeed, any connection between VitD metabolism and body composition is strongly affected by serum levels of 25-hydroxyvitamin D (25OHD). 25OHD levels in turn are primarily determined by environmental factors, mainly UV-B exposure, diet, and VitD supplementation. Thus the comparatively minor effects of genetic polymorphisms on VitD metabolism are easily obscured by variance in such environmental confounders. The simultaneous assessment of 25OHD levels and minimization of the above environmental factors would be ideal for studies conducted in this area. To our best knowledge this had not been the case in previous research.

In a previous analysis of the same adult population we have found no significant correlation between free and bioactive 25-hydroxyvitamin D (f-25OHD, b-25OHD) and BMI with the minimization environmental confounders [25]. In this present study, we aimed to identify genetic polymorphisms of VitD metabolism that are associated with BMI irrespective of serum 25OHD levels. Such polymorphisms hypothetically could alter the function of VDR or enzymes involved in the activation of 25OHD or the elimination of calcitriol. Genetic differences in other genes of VitD metabolism could hypothetically lead to changes in body composition independent of serum 25OHD levels, for example via changes in autocrine/paracrine signaling in adipose tissue.

In this present paper, we investigated the relationship between BMI and genetic polymorphisms of major enzymes of VitD metabolism in a representative adult population with the minimization of any confounding environmental factors known to affect VitD metabolism. A significant association in this regard could reinforce the importance of VitD physiology in body weight regulation.

## Methods

### Subjects

A random and representative cross-sectional sample (*n* = 892) of Hungarian adults was surveyed in 2013. Detailed medical history, relevant lifestyle information and the results of physical examination were recorded. In order to maximize genetic effects of the polymorphisms related to VitD metabolism, we tried minimizing all potentially confounding environmental factors. Due to the location of Hungary, ambient solar UV-B radiation is insufficient for significant VitD synthesis between autumn and spring. Thus blood samples were taken within a one week interval, before the first two successive sunny days of the year, at the beginning of April. Participants with lifestyle factors that are recognized to affect VitD levels, such as the use of indoor tanning, recent (within 3 months) overseas travel, and VitD supplementation were excluded. Of the initially enrolled 892 subjects 223 fulfilled one or more of the aforementioned exclusion criteria. An additional 207 patients were excluded from the genetic analysis due to the unavailability of either relevant medical data or blood samples, which resulted in a final study population of 462 individuals. Subject characteristics are presented in Table 1. There was no significant difference between the original survey and the final study sample except for a slightly decreased female to male ratio in the latter.

**Table 1:** Characteristics of the full survey and the genetic substudy population

Parameter	Survey participants (*n* = 669)	Study population (*n* = 462)	*p*-value
Age (years ± SD)	49.62 ± 16.65	49.55 ± 16.63	0.87
Gender (n (%))			
Male	281 (42%)	228 (49%)	< 0.0001
Female	383 (58%)	234 (51%)	
Height (m ± SD)	1.69 ± 0.09	1.70 ± 0.1	0.97
Weight (kg ± SD)	74.9 ± 16.0	75.7 ± 15.9	0.90
BMI (kg/m^2^ ± SD)	25.8 ± 4.6	26.0 ± 4.5	0.88
Place of residence (n)			
Capital	153 (23%)	65 (14%)	0.47
City	309 (46.5%)	264 (57%)	
Village	202 (30.5%)	133 (29%)	
Occupation (n)			
Blue collar	252 (38%)	179 (39%)	0.32
White collar	412 (62%)	283 (61%)	
t- 25(OH) D (nmol/L ± SD)	41.8 ± 20.9	37.4 ± 17.5	0.0002
b-25(OH) D (nmol/L ± SD)	4.8 ± 4.3	3.7 ± 2.2	< 0.0001
f- 25(OH) D (pmol/L ± SD)	11.8 ± 10.3	9.2 ± 5.3	< 0.0001
DBP (mg/L ± SD)	303.8 ± 99.3	293.1 ± 107.6	0.47
PTHi (pg/ml ± SD)	25.3 ± 16.3	26.7 ± 18.4	0.20

*Abbreviations*: *b-25OHD* bioactive 25-hydroxyvitamin D, *BMI* Body Mass Index, *DBP* vitamin D binding protein, *f-25OHD* free 25-hydroxyvitamin D, *t-25OHD* total 25-hydroxyvitamin D, *PTHi* intact parathormone

### Data collection

The participants’ medical history, relevant lifestyle information, anthropometric data and vital signs were recorded and blood samples were taken by the family physician during a single visit. Blood sampling was carried out under fasting conditions maintaining standardized procedures. Serum levels of total 25OHD (t-25OHD), vitamin D binding protein (GC/DBP) and albumin were measured. b-25OHD levels were calculated using C.E. Powe’s method [26], while f-25OHD levels were determined by D.D. Bikle’s formula [27]. Detailed description of the analytical methods used in this study can be found in our previous work [25].

### Genotyping

We analyzed the genetic variations of the main proteins involved in VitD metabolism. As the influence of UV-B exposure was minimized by the study design, we omitted genes linked to skin pigmentation. Accordingly, the selected proteins were glutamine-dependent NAD(+) synthetase (NADSYN1), vitamin D 25-hydroxylase (CYP2R1), DBP (GC), vitamin D 24-hydroxylase (CYP24A1), and vitamin D receptor (VDR).

Candidate SNPs rs4588, rs7041 (GC); rs2209314, rs927650, rs4809959 and rs2762939 (CYP24A1) were selected on the basis of their previously established correlation with t-25OHD levels. In addition we performed pairwise tag SNP selection using the HapMap CEU population data (accessed in Jan 2016) with a threshold for linkage disequilibrium at r2 = 0.8 for SNPs with a minor allele frequency of at least 0.2. The resulting tag SNPs were: rs11023374, rs10500804, rs1993116 for CYP2R1; rs222054, rs17467825 for GC; rs6022999, rs4809960, rs2181874, rs2585428, rs2244719, rs2762941 rs3787555 for CYP24A1 rs7935125 for NADSYN1 and rs1544410, rs3890733, rs7302235 and rs2853564 for VDR. DNA was obtained from EDTA-venous blood samples by use of a commercial DNA extraction kit (High Pure PCR, Roche, Meylan, France). Genotyping was undertaken at Innsbruck Medical University’s Sequencing and Genotyping Core Facility using a MASSarray Analyzer 4 (Sequenom Inc., San Diego, California, USA).

### Statistical analysis

All descriptive data are presented as percentages for nominal variables, and mean ± 1 standard deviation (SD) for scale variables. Statistical tests of normality and inspection of Q-Q plots and histograms were used to test normality for continuous variables. Haplotype block analysis was used for all selected SNPs to assess linkage disequilibrium. We performed Chi-square tests to exclude deviation from the Hardy-Weinberg equilibrium. For the investigation of unadjusted association between the investigated SNPs and BMI, a dominant model was applied by grouping individuals carrying at least one copy of a minor allele into a single group. Then BMI levels between these groups were compared using two-sample t-tests. To correct for multiple testing, we applied the Benjamini-Hochberg procedure to control the false discovery rate to be less than 15%. A corrected two sided *p*-value < 0.05 was viewed as statistically significant.

SNPs that were significantly associated with BMI were entered in a single multivariate linear model with BMI as the dependent, the SNPs and DBP and t-, f-25OHD levels as independent variables. Due to the strong correlation (r2 = 0.981) between f-25OH- and b-25OH-vitamin D levels, the latter was omitted from this final model. Statistical analysis was performed with the use of Haploview [28] and SPSS for Windows (version 21, SPSS Inc., Chicago, IL, USA).

## Results

Anthropometric characteristics and laboratory data of the full survey sample (*n* = 669) and the 462 participants included in the present genetic analysis are presented in Table 1. There are two notable intergroup differences that have to be mentioned. First, serum 25OHD, b-25OHD, and f-25OHD were lower in our sample compared to the original population related to the exclusion of participants with a history of VitD supplementation or other confounding environmental factors. VitD levels were skewed, and over 90% of the participants were vitamin D insufficient (defined as serum 25OHD below 75 nmol/L) and 77% were vitamin D deficient (25OHD below 50 nmol/L) even in the original survey sample. Second, we found a lower proportion of women in the current study population compared to the full sample related to the higher frequency of VitD supplementation among women, especially among those over the age of 50. Age, anthropometric parameters, place of residence and occupation were similar in the study and the full survey sample. Similarly to the findings in our previous report on the full survey sample [29], BMI showed no association with free or bioactive 25OHD levels. Average BMI was in the overweight category and was comparable to the values reported in the 2014 Hungarian Diet and Nutritional Status Survey [30].

All investigated SNPs were in Hardy-Weinberg equilibrium. With the exception of rs4588 and rs7041 (r2: 0.83), none of the SNPs were in linkage disequilibrium. Table 2 shows univariate associations between the analyzed SNPs and BMI levels. After correction for multiple testing with the Benjamini-Hochberg method, significant associations were retained for the following SNPSs: rs11023374 (CYP2R1), rs2853564, rs7302235 (VDR).

**Table 2:** BMI levels in subgroups defined by the investigated genotypes

		Allele		BMI (kg/m^2^)		
Gene	SNP	ref.	var.	ref. homo-zygote.	minor allele carriers	p
**NADSYN1**	rs7935125	C	A	25.3 ± 4.2	26.0 ± 4.5	0.31
**GC**	**rs17467825**	**A**	**G**	**26.4** ± 4.6	**25.6** ± 4.4	**0.045**
	rs222054	C	G	25.9 ± 4.5	26.1 ± 4.6	0.76
	**rs4588**	**G**	**T**	**26.4** ± 4.6	**25.6** ± 4.4	**0.048**
	rs7041	A	C	25.4 ± 4.1	26.2 ± 4.6	0.13
**CYP2R1**	rs1993116	A	G	26.5 ± 4.5	25.9 ± 4.5	0.295
	rs10500804	T	G	26.4 ± 4.6	25.8 ± 4.5	0.15
	**rs11023374**	**T**	**C**	**26.5** ± 4.4	**25.3** ± 4.4	**0.006**^**a**^
**CYP24A1**	rs4809959	A	G	25.9 ± 4.1	26.0 ± 4.6	0.89
	rs927650	T	C	26.0 ± 4.4	25.9 ± 4.5	0.89
	rs4809960	T	C	26.1 ± 4.4	25.8 ± 4.7	0.49
	rs2209314	T	C	25.9 ± 4.4	26.2 ± 4.8	0.41
	rs6022999	A	G	26.1 ± 4.3	25.8 ± 4.8	0.54
	rs2181874	G	A	25.8 ± 4.3	26.2 ± 4.8	0.37
	rs2585428	C	T	25.9 ± 4.1	26.0 ± 4.7	0.72
	**rs3787555**	**C**	**A**	**26.4** ± 4.4	**25.5** ± 4.6	**0.038**
	rs2762939	G	C	25.7 ± 4.4	26.4 ± 4.6	0.11
	rs2244719	C	T	26.1 ± 4.0	25.9 ± 4.7	0.74
	rs2762941	G	A	25.7 ± 4.3	26.1 ± 4.6	0.37
**VDR**	rs1544410	G	A	26.1 ± 4.4	25.9 ± 4.6	0.64
	rs3890733	C	T	26.3 ± 4.8	25.8 ± 4.3	0.20
	**rs2853564**	**G**	**A**	**24.5** ± 4.3	**26.2** ± 4.5	**0.007**^**a**^
	**rs7302235**	**T**	**C**	**25.5** ± 4.2	**26.6** ± 4.9	**0.017**^**a**^

Univariate associations between SNPs and BMI were analyzed using two-sample t-tests

^a^ marks association that remained significant after FDR correction

*Abbreviations*: *CYP24A1* vitamin D 24-hydroxylase gene (cytochrome P450 family 24 subfamily A member 1), *CYP2R1* vitamin D 25-hydroxylase gene (cytochrome P450 family 2 subfamily R member 1), *GC* vitamin D binding protein, “group-specific component” gene, *NADSYN1* glutamine-dependent NAD(+) synthetase, *VDR* vitamin D receptor

Bold text highlights where *p* < 0.05

All three SNPs were combined into a multivariable linear model controlling for serum DBP, t-25OHD and f-25OHD levels. As it can be seen in Table 3, rs2853564 and rs11023374 remained significantly associated with BMI, while rs7302235 became a non-significant determinant. The two significant SNPs explained 3.2% of the BMI variance (1.6% each). The effect size between the most and least favorable haplotypes is nontrivial (0.70–1.56 kg/m2).

**Table 3:** Association between BMI and selected genotypes adjusted for vitamin D levels and DBP

Parameter	B	Std. error	*P*-value	Explained variance (eta-square)
rs11023374	1.080	0.444	0.015	1.6%
rs2853564	−1.557	0.633	0.014	1.6%
rs7302235	−0.699	0.453	0.124	0.6%
calculated genetic effect			NA	3.2%
t-25OH-vitamin D	0.019	0.082	0.858	0%
f-25OH-vitamin D	0.296	0.224	0.751	0%
DBP	−0.016	0.005	0.002	2.5%

Multiple linear regression with BMI as dependent and SNPs with significant univariate association (rs11023374, rs2853564, rs7302235) and DBP, t-25OHD, f-25OHD as covariates

*Abbreviations*: *b-25OHD* bioactive 25-hydroxyvitamin D, *DBP* vitamin D binding protein, *f-25OHD* free 25-hydroxyvitamin D, *t-25OHD* total 25-hydroxyvitamin D

eQTL data was explored using the multi-tissue eQTL dataset (v8), from the GTEx database (https://www.gtexportal.org/home/datasets). While neither SNPs has a known eQTL effect in subcutaneous or visceral adipose tissue, both rs11023374 and rs2853564 were found to increase protein expression in skeletal muscle (*p* values of 0.00038 and 0.01 respectively). Genomic reference data from the 1000 Genomes Project [31] suggested a possible linkage disequilibrium between rs11023374 and rs10832313 (D’: 0.9889 across all sample populations).

## Discussion

In the current study we have identified two polymorphisms in the VDR and CYP2R1 genes that are associated with BMI independently of serum 25OH-, free-, or bioactive VitD levels. There is a possible linkage disequilibrium between one of these SNPs and another variant that have been postulated but not definitively proven to have an effect on body composition. Both rs2853564 and rs11023374 are intron variants, with no relevant data in the literature on their function. The effect size of these polymorphisms on BMI is considerable and thus, these or other SNPs in linkage might have a potential role in transcription regulation. This theory is further supported by available eQTL data which suggests increased transcription associated with both SNPs in skeletal muscle.

The ever growing epidemic of obesity is at the forefront of discussions of public health in the western world. Though it is considered mainly a result of lifestyle choices and medical comorbidity, more and more emphasis is being placed on the significance of underlying genetic susceptibility [1, 32]. Some rare monogenic causes of obesity have been known for decades and others have been identified in the past 10–15 years [33, 34]. More importantly, however, complex genetic polymorphisms precipitating common obesity have been investigated in recent years. On top of an increasingly obesogenic environment, these genetic factors are likely to play a substantial role in the obesity epidemic, particularly in case of childhood obesity. Familial and twin studies suggest heritability between 60 and 90% for overweight, while the authors of a large meta-analysis based on recent GWAS and Metabochip studies estimate that around 21% of BMI variation can be accounted for by common genetic variations [1, 35–37]. In recent years, over a hundred genetic loci have been identified as having a possible association with BMI. These variations are in part proven but more commonly hypothesized to affect one or more of the many levels of body-weight regulation and energy homeostasis. Among these known genetic polymorphisms, variants of the fat mass and obesity (FTO) gene seem to have the uppermost effect and possible therapeutic implications [38].

Vitamin D is one of the oldest members of the steroid-hormone family [39]. In the past 10–20 years there has been an exponential interest in its physiologic action and pathological significance. This resulted in the recognition of extensive effects well beyond its previously established role in bone and mineral homeostasis [40]. There is an irrefutable relationship between obesity and VitD deficiency. BMI over 30 kg/m^2^ is a well-established risk factor for VitD insufficiency/deficiency [9]. A few plausible explanations include sequestration in adipose tissue, decreased physical activity and sun exposure in overweight and obese people, decreased 25- hydroxylation capacity of the liver related to fatty degeneration and the influence of certain adipokines like leptin and IL-6. The converse of this association also seems to be plausible. Vitamin D deficiency may worsen metabolism, increases insulin resistance and the risk of both type 1 and type 2 diabetes [41–43]. VitD and calcium supplementation is found to decrease the amount of visceral fat, and cholecalciferol substitution alone was shown to improve insulin resistance [44, 45]. It seems that calcitriol, the active form of VitD might play an influential role in the regulation of the adipose tissue as an endocrine organ. This is also underpinned by the presence of both VDR, and the enzymes 1α-, and 24-hydroxilase in fat cells [46]. In vitro studies showed VDR having an effect on the maturation of adipose cells via the PPARγ signalling pathway [47]. The influence of VitD on adipokine production was also demonstrated in numerous trials both in vitro and in vivo [22, 48].

Based on the above mentioned findings, a number of studies have examined the relationship between obesity and/or metabolic syndrome and genetic variations in different proteins of VitD metabolism. Results regarding VDR and obesity are numerous but conflicting [20–24, 49–52]. Population size, homogeneity, ethnicity, the SNPs examined and the obesity measures used in these studies vary widely. Results are also heterogeneous ranging from obesity being associated with multiple VDR SNPs to no significant association being found at all. Investigation of other members of VitD metabolism is scarcely undertaken. In a single study [53], the rs17467825 polymorphism of DBP correlated with fat mass and body fat percentage among 1873 adults. The two largest studies to date analysing several SNPs of multiple genes of VitD metabolism [18, 19] also present conflicting results. Vimaleswaran et al. have investigated 100 tagging SNPs in the DHCR7, CYP2R1, VDBP, CYP27B1, CYP27A1, CYP24A1, VDR and RXRG genes in the 1958 British birth cohort and GIANT consortium (5224 and 123,865 participants respectively). A single SNP; rs2296239 in CYP24A1 showed association with just one obesity trait (waist-hip ratio), and even this association was significant in only one of the cohorts. The authors thereby concluded that vitamin-D genetics are unlikely to play a significant role in obesity [18]. Dorjgochoo et al. have examined the relationship between BMI and 198 SNPs in the CYP27A1, CYP27B1, CYP24A1, CYP2R1, GC and VDR genes in a sample of nearly 7000 Chinese women. Two SNPs (rs2248359 in CYP24A1 and rs10832313 in CYP2R1) showed associations with BMI, however, this did not remain significant after accounting for multiple testing. The authors concluded that the evidence does not support a strong causal connection between VitD deficiency and obesity, and called for additional studies in other populations [19].

The two SNPs (rs2853564- VDR, rs11023374- CYP2R1) identified in our work have not previously been linked to obesity phenotypes. However, rs11023374 is suggested to be in linkage disequilibrium with rs10832313 that was found to be nominally associated with BMI by Dorjgochoo et al. As rs11023374 and rs10832313 are both intron variants, linkage disequilibrium with an exonic variant or a potential role of these genetic regions in transcription regulation of CYP2R1 could explain their effect on BMI. The effect of rs2853564 could potentially also be mediated through tissue specific transcription regulation of VDR. Available eQTL data hint towards skeletal muscle as a potential site of action of these SNPs. A direct effect on muscle mass or a role in skeletal muscle-adipose tissue crosstalk could be hypothesized.

Our study has a number of strengths. First, unlike most previous works on the topic that concentrated mainly on variations in the VDR gene, SNPs in all relevant proteins and enzymes were examined. Second, it was conducted on a representative and random sample of Hungarian adults, while most previous works used biased sampling that is potentially less suited for large-scale genetic testing. Third, we used a selection method that minimized environmental confounders, and measured and corrected our results for serum VitD levels in all participants. VitD metabolism is heavily affected by sunlight exposure, food intake and VitD supplementation. Effects of a genetic origin are most likely comparatively small and easily obscured by such environmental differences. The numerous contradictory findings of previous studies might partly result from the inter- and intra-study heterogeneity of environmental determinants and VitD levels of participants. Our notion is that testing aimed at these genetic effects should take this into consideration.

Our study has a number of limitations that should be acknowledged. A large number of participants had been excluded from the analysis due to environmental confounders, which made the final study sample smaller than originally planned. As anthropomorphic data were collected during routine GP visits, only BMI was used instead of more sophisticated obesity measurements. While tag SNPs were selected to cover a large number of common variants within the candidate genes, they still represent only a fraction of the potential variation and full gene coverage was not achieved. Additionally, because our study focused only on genes involved in VitD metabolism, other genetic loci that could affect the vitamin D-obesity connection were not examined. While our study sample was representative of the adult population of Hungary, replication of these results in other population samples is needed to further validate them.

## Conclusion

In our population sample, two SNPs showed a significant association with BMI that was not affected by serum t-, f-, or b-25OHD levels. One of these SNPs is possibly in linkage with another variant previously suggested but not definitively proven to be associated with BMI. While the direct effect of vitamin D-related genetic differences on obesity or BMI appear to be small, these results further emphasize the proposed role of vitamin D in regulating body composition [54, 55], and warrant further investigations into the molecular and genetic background of this association.

## Data Availability

The dataset used and analysed during the current study is available in the Mendeley Data repository: Bakos, Bence; Szili, Balázs; Szabó, Boglárka; Horváth, Péter; Kirschner, Gyöngyi; Kósa, János; Toldy, Erzsébet; Lakatos, Péter; Takács, István (2020), “Genetic variants of VDR and CYP2R1 affect BMI independently of serum vitamin D concentrations”, Mendeley Data, V2, doi: 10.17632/h34239mrgy.2 eQTL data was explored using the multi-tissue eQTL dataset (v8), from the GTEx database: https://www.gtexportal.org/home/datasets. Tag SNP selection was performed using the HapMap CEU population data: https://ftp.ncbi.nlm.nih.gov/hapmap/ To investigate potential linkage disequilibrium with previously reported SNPs, we used reference datasets from the 1000 Genomes Project accessed through LDlink. ftp://ftp.1000genomes.ebi.ac.uk/vol1/ftp/phase3 ftp://ftp.1000genomes.ebi.ac.uk/vol1/ftp/release/20130502/ https://ldlink.nci.nih.gov/ The accession numbers corresponding to other datasets obtained from NCBI and used in this study can be found in Table 2.

## Abbreviations

VitD: Vitamin D
25OHD: 25-hydroxyvitamin D
b-25OHD: Bioactive 25-hydroxyvitamin D
CYP24A1: Vitamin D 24-hydroxylase gene (cytochrome P450 family 24 subfamily A member 1)
CYP2R1: Vitamin D 25-hydroxylase gene (cytochrome P450 family 2 subfamily R member 1)
DBP: Vitamin D binding protein
f-25OHD: Free 25-hydroxyvitamin D
FTO: Fat mass and obesity gene
GC: Vitamin D binding protein, “group-specific component” gene
NADSYN1: Nicotinamide adenine dinucleotide synthetase
t-25OHD: Total 25-hydroxyvitamin D
VDR: Vitamin D receptor
